# Corrigendum: A Probabilistic Framework for Decoding Behavior From *in vivo* Calcium Imaging Data

**DOI:** 10.3389/fncir.2020.629162

**Published:** 2020-12-11

**Authors:** Guillaume Etter, Frederic Manseau, Sylvain Williams

**Affiliations:** Douglas Mental Health University Institute, McGill University, Montreal, QC, Canada

**Keywords:** calcium imaging, decoding, bayesian inference, hippocampus, spatial coding

In the original article, there were errors in several equations. Some terms were flipped or omitted.

Corrections have been made to *Results, Extracting Probability Values in a Bayesian Context, Equations 1 and 2*, and *Results, Decoding Behavior Using Activity From Multiple Neurons, Equations 7 and 8*.

The Corrected Equations are Shown Below:

Equation 1:

$$\begin{array}{l} P\text{(A)}=\frac{time\;active}{total\;time} \end{array}$$

Equation 2:

$$\begin{array}{l} P\text{(}{\text{S}}_{i}\text{)}=\frac{time\;in\;state\;i}{total\;time} \end{array}$$

Equation 7:

$$\begin{array}{l} P\;\left(S|A\right)=\text{exp}\left[\overset{N}{\underset{k=1}{\sum }}\log\left(1+\frac{P\left(A_{k}|S\right)\;\times P\left(S\right)}{P\left(A_{k}\right)}\right)-1\right] \end{array}$$

Equation 8:

$$\hat{y}=\;arg\;max\;exp[\sum_{k=1}^{N}\log(1+\frac{P(A_{k}|S)\times P(S)}{P(A_{k})})-1]$$

The authors apologize for these errors and state that they do not change the scientific conclusions of the article in any way. The original article has been updated.

